# Trends in Glucagon-Like Peptide-1 Receptor Agonist Social Media Posts Using Artificial Intelligence

**DOI:** 10.1016/j.jacadv.2024.101182

**Published:** 2024-08-28

**Authors:** Aamir Javaid, Sruthika Baviriseaty, Rehan Javaid, Ayah Zirikly, Harshita Kukreja, Chang H. Kim, Michael J. Blaha, Roger S. Blumenthal, Seth S. Martin, Francoise A. Marvel

**Affiliations:** a Ciccarone Center for the Prevention of Cardiovascular Disease, Division of Cardiology, Department of Medicine, Johns Hopkins University School of Medicine, Baltimore, Maryland, USA; b Digital Health Innovation Laboratory, Johns Hopkins University School of Medicine, Baltimore, Maryland, USA; c Whiting School of Engineering, Department of Computer Science, Johns Hopkins University, Baltimore, Maryland, USA; d Computer Science Department at New York University, NYU Graduate School of Arts & Science, New York, New York, USA

**Keywords:** artificial intelligence, GLP-1RA, natural language processing, ozempic, social media

## Abstract

**Background:**

Glucagon-like peptide-1 receptor agonists (GLP-1RAs) have surged in popularity in recent years, with discussions about their on-label and off-label use spilling into the public forum. No study has analyzed online discussions about GLP-1RAs.

**Objectives:**

The purpose of this study was to analyze perceptions of GLP-1RAs on social media.

**Methods:**

We analyzed GLP-1RA-related posts on Reddit between May 28, 2013, and June 1, 2023. All posts were identified that included generic or brand names of GLP-1RAs. Post volume on Reddit was compared to search interest on Google over time. An artificial intelligence (AI) pipeline consisting of a semi-supervised natural language processing model (Bidirectional Encoder Representations from Transformers [BERT]), a dimensionality reduction technique, and a clustering algorithm was used to cluster posts into related topics. Discussion sentiment was classified using a pretrained BERT model and assessed qualitatively.

**Results:**

14,390 GLP-1RA-related Reddit posts by 8,412 authors were identified. Ninety-four percent of posts were created after 2021, consistent with search interest trend on Google. We used the AI model to categorize posts into 30 topics which were hierarchically grouped by the model based on shared content. Posts were identified among communities for individuals with diabetes and obesity, as well as for diseases without a Food and Drug Administration-approved indication. Most posts had a negative sentiment using the pretrained model, acknowledging the pretrained model is at risk for misclassifying posts.

**Conclusions:**

AI can generate insights on perceptions of GLP-1RAs on social media. Common themes included success stories of improving diabetes and obesity management, struggles with insurance coverage, and questions regarding diet, side effects, and medication administration.

Glucagon-like peptide-1 receptor agonists (GLP-1RAs) have demonstrated clinical benefits for a range of pathologies, among them type-2 diabetes, obesity, and cardiovascular diseases.^1^^,^^2^ Demand for these medications is surging, with some suggesting the exponential growth of online searches relative to prescription patterns as an indication that demand is partially driven by patient interest.^3^ While reasons for the popularity of GLP-1RAs have been purported, no studies have systematically examined trends in social media posts on this medication class.

Artificial intelligence (AI), a branch of computer science that aims for computers to engage in human-like thought processes, provides an avenue to identify patterns nonlinearly across vast quantities of data.^4^ Natural language processing (NLP) is a subset of AI that interprets textual data.^5^ Besides recent acclaim for its use in chatbots and large language models, NLP has also been used to analyze social media discussions on medical topics on Reddit, a free discussion-based platform with 52 million daily active users, approximately 430 million monthly users, and more than 30 billion views per month.^6^ Analyzing social media discussions with AI has the potential to elucidate patient experiences, priorities, questions, challenges, and areas of controversy, identify interested stakeholders, and gauge medication demand and use patterns worldwide. We aimed to use NLP to analyze social media posts on GLP-1RAs.

## Methods

This qualitative study used online social media posts in the public domain and did not directly involve human participants. Hence, this study was exempt from need for ethical review and informed consent.

### Data set and search strategy

**Figure 1:**
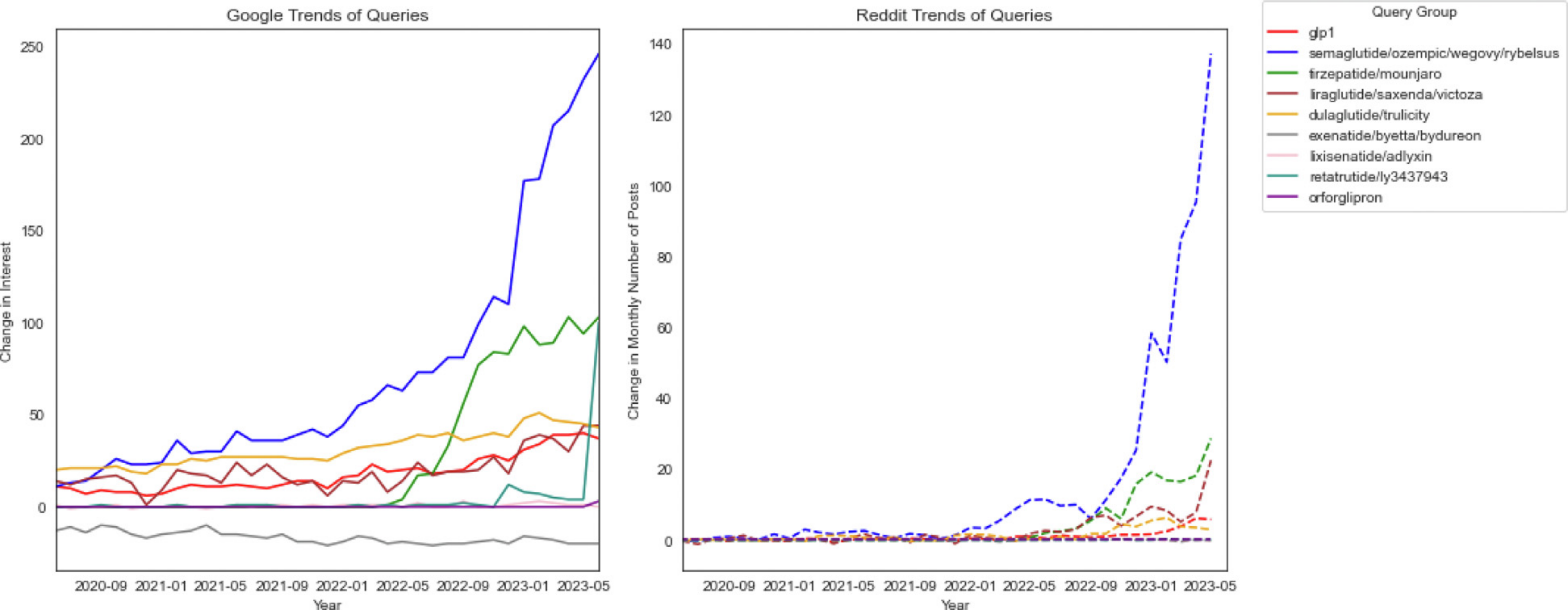
Interest in GLP-1RA Search Query Groups on Google Trends (Left) and Change in Monthly Number of Posts by Unique Users on Reddit (Right) From 2020 to 2023 Queries for brand names were grouped under the shared generic name. GLP-1RA = glucagon-like peptide-1 receptor agonist.

Methods were adapted from a recent study by Somani et al.^6^ Data from Reddit were collected using the Reddit application programming interface between May 30, 2013, and June 1, 2023. Posts were identified by searching for variations of GLP-1RAs (GLP-1, GLP1, GLP-1RA, GLP1RA) and the generic and brand names of specific GLP-1RAs: dulaglutide, Trulicity, exenatide, bydureon BCise, Byetta, semaglutide, Ozempic, Rybelsus, orforglipron, liraglutide, Victoza, Saxenda, lixisenatide, Adlyxin, tirzepatide, Mounjaro, retatrutide, and LY3437943. The same search terms were used on Google Trends. Change in search interest on Google was contrasted to change in post volume on Reddit in Figure 1, with Reddit data undergoing smoothing using a 6-month moving average to enhance clarity in the overall trend.

### Topic modeling

Raw text posts were collected from Reddit and preprocessed for analysis using BERTopic, as previously described.^6^^,^^7^ Briefly, 30 topics were identified by spectral clustering, in which similar discussions are grouped. We experimented with different numbers of topics ranging by units of 10 from 10 to 100; in this case, we chose 30 topics to provide the best balance of achieving topic granularity without over-compartmentalizing into topics whose shared characteristics were difficult to understand upon human review. These were further clustered into overarching themes. Three themes were selected by optimizing the Silhouette coefficient and Davies-Bouldin index,^8^^,^^9^ as previously described.^6^ Topics within the 3 themes were reviewed by humans and a label was assigned based on shared content. Some of the themes had a diversity of topics that could not all be encompassed with a 1- or 2-word label; in these cases, commonalties shared by the majority of topics were used to assign a label. The details of all topics and themes can be found in the online materials.

### Sentiment analysis

To assess the sentiment of posts, we used a pretrained BERT model called RoBERTa as previously described to classify posts using a scale from −1 (negative) to +1 (positive) with 0 being neutral.^6^^,^^10^ A random sample of 9 posts classified positive and 9 posts classified negative by RoBERTa were reviewed by a human clinician to try to understand features used by the model to assign post sentiment. These posts were also analyzed using the OpenAI gpt-3.5-turbo model using the prompt: “You will be provided with a text post, and your task is to classify its sentiment as positive, neutral, or negative.” Analysis of the entire data set with ChatGPT was not possible due to financial constraints.^11^

### Statistical analysis

We described discussion characteristics using mean ± SD. Analysis was performed using the Python programming language, version 3.7.3 and multiple key libraries: scikit-learn, version 1.1.1; BERTopic, version 0.11.0; transformers, version 4.20.1; and matplotlib, version 3.5.2.^12^ This work was carried out at the Advanced Research Computing at Hopkins (ARCH) core facility (rockfish.jhu.edu), which is supported by the National Science Foundation (NSF) grant number OAC1920103. Code used for topic modeling and analysis is available online.^13^

## Results

A total of 14,390 Reddit posts were curated from 8,412 users. 13,521 (94%) posts were created after 2021. To better visualize and compare interest in medications over time, we grouped brand name medications under the generic name in Figure 1. The figures focus on the period from 2020 to 2023, highlighting recent trends in online query dynamics. Interest by individual search query is available in the supplement (Supplemental Figures 1 and 2). The rate of post volume change for each drug on Reddit increased similarly to search interest on Google Trends over time. Semaglutide-related terms had the most interest (“Ozempic,” followed by “Rybelsus,” “Wegovy,” and “semaglutide”) followed by tirzepatide-related terms (“Mounjaro,” then “tirzepatide”). In general, brand names were more popular than generic names. Retatrutide, a triple-action GLP-1/glucose-dependent insulinotropic polypeptide/glucagon agonist, which is not yet commercially available, had almost no posts on Reddit but was the third most searched query on Google by the end of the study period.

**Figure 2:**
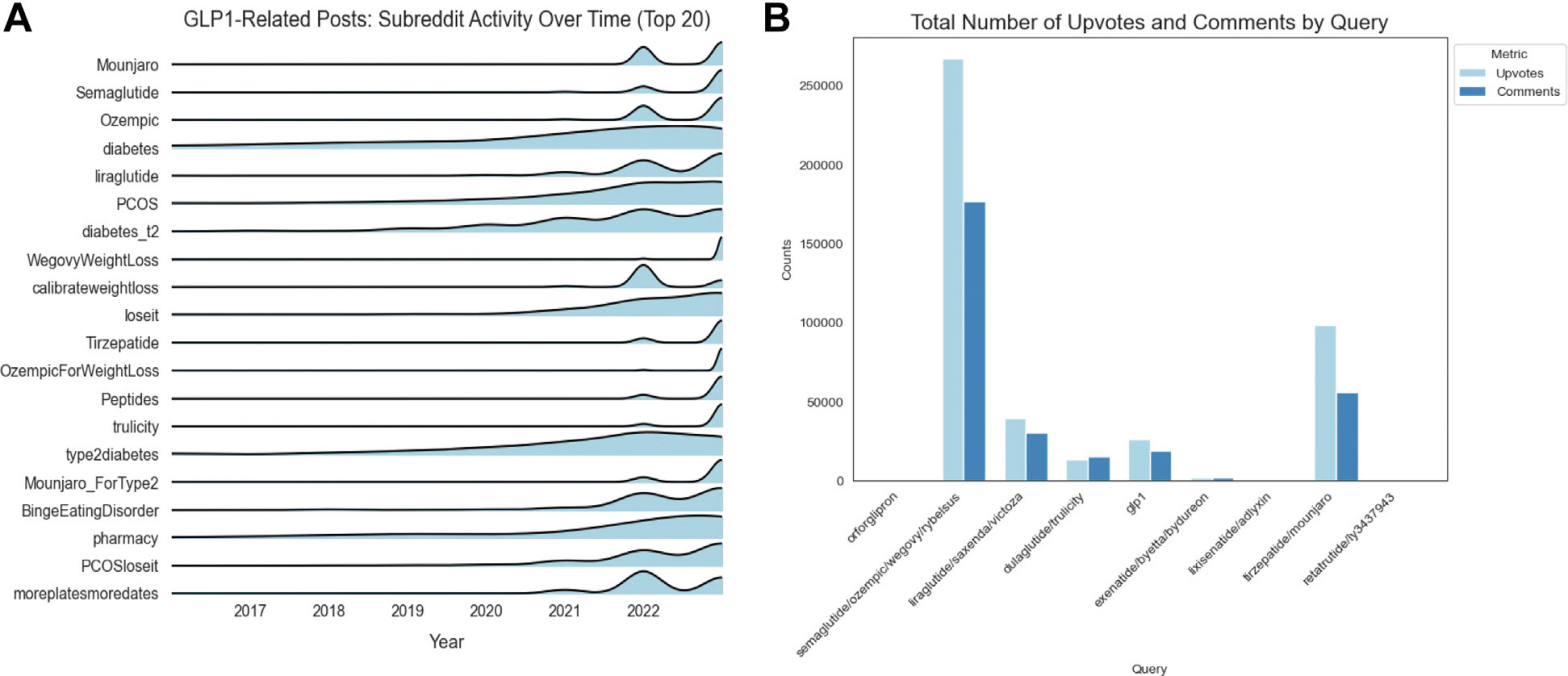
Trends in Reddit Communities and Post Engagement (A) Post activity over time in the top 20 most posted subreddits (reddit communities). Notable categories include medication specific (“Ozempic,” “Mounjaro”), weight loss (“Loseit,” “OzempicforWeightLoss”), addiction (“BingeEatingDisorder,” “Stopdrinking”), women’s health (“PCOS,” “Menopause”), wellness/biohacking (“Longevity,” “Moreplatesmoredates,” “Testosterone”), bodybuilding (“SemaglutideandGains”), and science (“Medicine,” “EverythingScience”). (B) Posts, upvotes, and comments by search query, grouped by shared generic name. PCOS = polycystic ovarian syndrome.

Figure 2A demonstrates activity trends in the 20 subreddits (Reddit communities in which posts are shared) with the highest number of posts containing our search queries. Subreddit “r/Mounjaro” had the most posts, followed by “r/Semaglutide” and “r/Ozempic,” despite the most popular search terms being for semaglutide-based drugs. While diabetes-related subreddits (“r/diabetes,” “r/type2diabetes”) had the most activity prior to 2021, medication-specific subreddits have since overtaken them in popularity. Figure 2B demonstrates the total number of posts, upvotes, and comments for each search query group, with statistics for individual queries in the supplemental results (Supplemental Figure 3).

**Figure 3:**
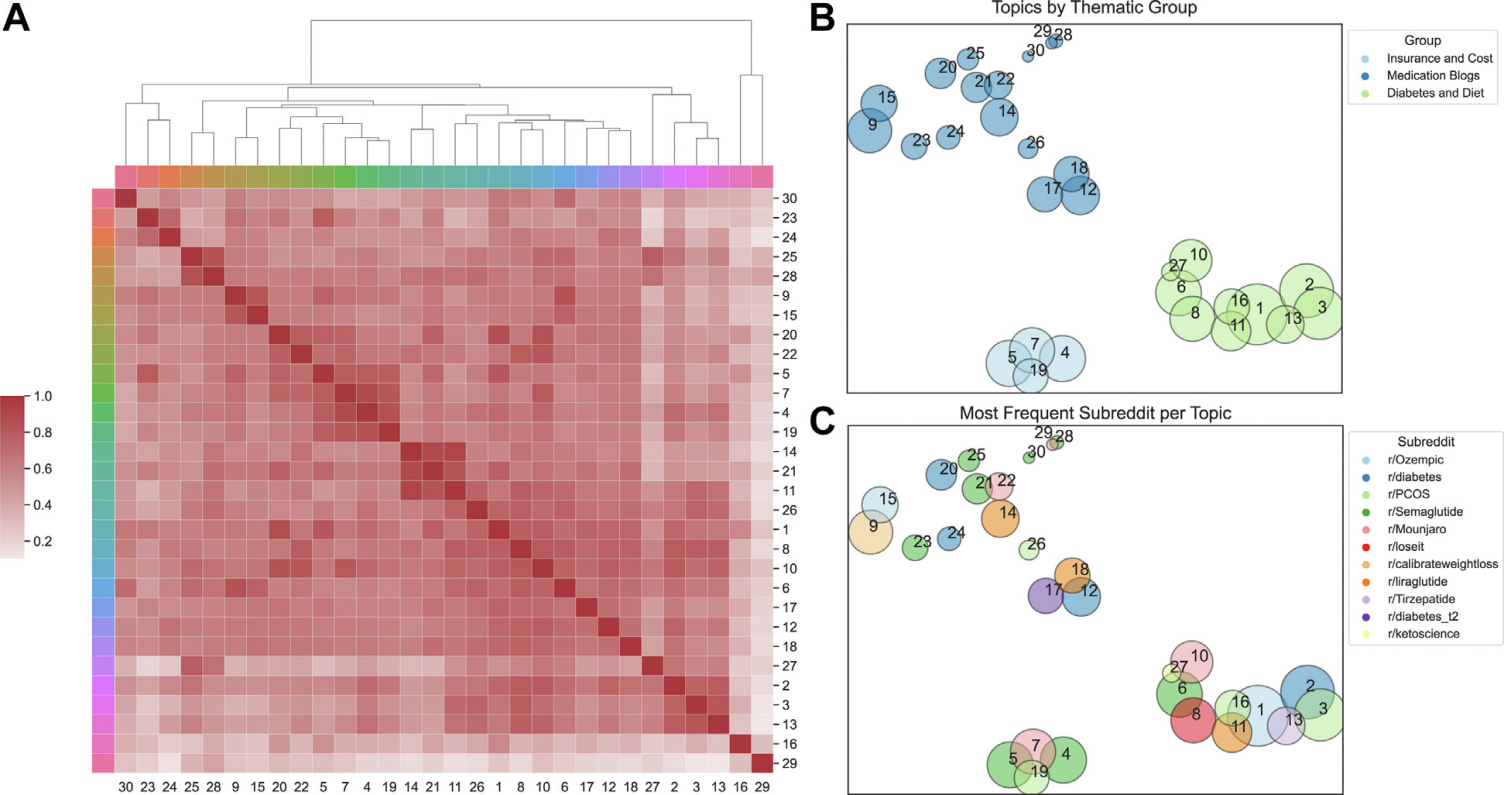
(A) Similarity Matrix of 30 AI-Generated Topics From Reddit Posts With Dendrogram of Hierarchical Relationships Between Topics The Y-axis represents the depth of the hierarchical tree corresponding to each node in the tree. Heatmap represents relative similarity of content between topics. (B and C) Spatial representation of the 30 extracted topics filtered by: (B) 3 Overarching themes, (C) Most frequent subreddit for each topic. AI = artificial intelligence.

Our AI pipeline identified 30 topics among the posts (Table 1) which were hierarchically clustered to identify 3 overarching themes: 1) insurance and cost, 2) medication-related blogs, and 3) diabetes/diet. Insurance and cost included terms such as “prior auth,” “coverage,” “denied,” and “savings.” Medication-related blogs involved individuals who were taking a specific medication and wanted to share their personal experiences or questions. Common themes were side effects (“nausea,” “fullness”), medication storage and administration (“pens,” “needles,” “fridge,” “injection”), and weight loss progress. The third group included diabetes-related terms (“a1c,” “insulin”), diet-related terms (“ketogenic,” “calories,” “binge eating”), and miscellaneous terms ranging from topics such as polycystic ovarian syndrome (PCOS), testosterone, television commercials, and reality television. Hierarchical representation of these topics is shown in Figure 3A with a similarity matrix heatmap demonstrating the relation between AI-generated topics. A spatial representation of the 30 topics is shown filtered by overarching theme (Figure 3B), the most frequent subreddit per topic (Figure 3C).

**Table 1:** Thirty AI-Generated Topics and Representative Terms for Each Topic Derived From 14, 390 Reddit Posts

Topic	Representative Terms
1	ozempic, nausea, side, effects, week
2	a1c, insulin, glucose, blood, carbohydrate
3	eating, calories, binge, eat, food
4	insurance, denied, cover, coverage, covered
5	pharmacy, stock, wegovy, 2022, australian
6	semaglutide, appetite, b12, fat, liraglutide
7	coupon, pa, card, mounjaro, savings
8	wegovy, week, shot, nausea, weeks
9	compound, compounded, compounding, semaglutide, pharmacy
10	mounjaro, metformin, maintenance, 5 mg, sucralose
11	saxenda, liraglutide, day, lost, eat
12	trulicity, 75 mg, side, stomach, 75
13	pcos, testosterone, hair, birth, periods
14	saxenda, wegovy, switch, dose, shortage
15	tirzepatide, peptides, peptide, tirz, semaglutide
16	commercials, chantal, claudia, commercial, kim
17	rybelsus, 7 mg, 14 mg, 3 mg, pill
18	victoza, 8 mg, byetta, liraglutide, anyone
19	calibrate, program, received, ro, refund
20	mounjaro, ozempic, switching, mj, oz
21	saxenda, liraglutide, australia, ozempic, weekly
22	wegovy, mounjaro, switching, switch, mj
23	pen, pens, fridge, clicks, room
24	needle, bydureon, injection, inject, needles
25	glp, study, agonist, kaiser, cagrisema
26	placebo, phentermine, bupropion, naltrexone, scary
27	ketogenic, glp, glucagon, gip, bone
28	discussion, glp1, ra, names, agonists
29	lounge, chat, members, place, glp1_ozempic_wegovy
30	desire, anonymous, drink, alcohol, irb

AI = artificial intelligence.

The overall sentiment for nearly all posts was negative or neutral by RoBERTa, with statistics per subreddit and per search query in Supplemental Tables 1 and 2, respectively. There were no appreciable changes in topics or post sentiment over time due to most posts occurring over a brief time frame of 2 to 3 years. We randomly selected and reviewed 9 positive and 9 negative posts to try to interpret features used by the model to classify sentiment (Supplemental Tables 3 and 4). We also assessed the sentiment of these posts using OpenAI’s gpt-3.5-turbo model. Posts with discordant sentiment by RoBERTa and ChatGPT are shown in Supplemental Table 5.

## Discussion

This study leveraged social media to assess public perceptions about GLP-1RAs (Central Illustration). 14,390 posts from Reddit were independently clustered by an AI model into topics based on shared themes. Over 90% of Reddit posts occurred after the Food and Drug Administration approval for semaglutide to treat adults with obesity on June 4, 2021.^14^ While weight loss itself did not emerge as an overarching theme of the AI-clustered topics, many Reddit posts discussed weight loss in the context of personal experiences from individuals using or interested in using GLP-1RAs. Notable other topics included frustrations with insurance and cost, treatment success stories, side effects, medication storage and administration, diet, and A1c changes. The absence of posts about retatrutide on Reddit, a premarket medication which was not yet commercially available during the study period, as compared to Google searches may suggest that Reddit posts were largely by people actively taking or trying to obtain a prescription for GLP-1RAs, as most Reddit users cannot access retatrutide.

**Central Illustration:**
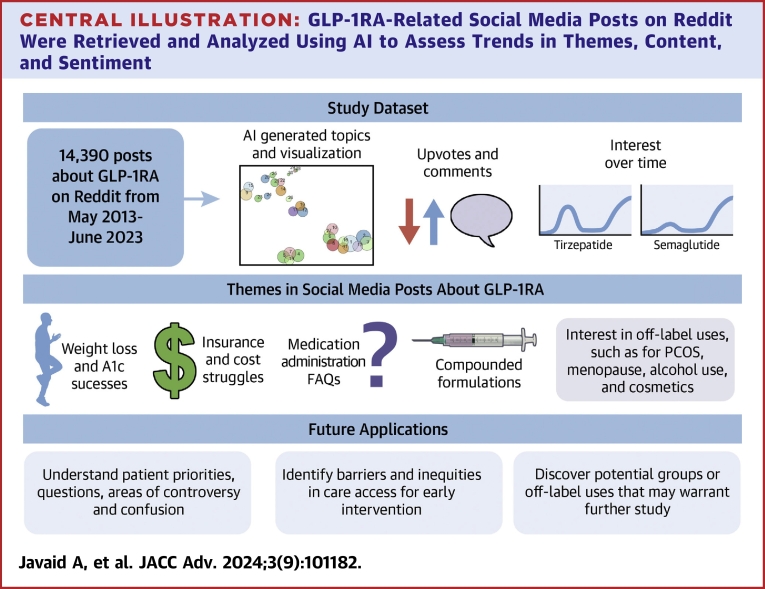
GLP-1RA-Related Social Media Posts on Reddit Were Retrieved and Analyzed Using AI to Assess Trends in Themes, Content, and Sentiment

The subreddits (Reddit communities) with the greatest number of posts mostly aligned with Food and Drug Administration-approved GLP-1RA indications of diabetes and weight loss (eg “r/Mounjaro,” “r/Semaglutide,” “r/Ozempic,” “r/liraglutide,” “r/diabetes,” “r/WegovyWeightLoss,” “r/loseit”). We also identified several nontraditional communities about topics such as addiction (“r/BingeEatingDisorder,” “r/stopdrinking”), women’s health (“r/menopause,” “r/PCOS”), testosterone deficiency (“r/trt”), and biohacking/wellness (“r/longevity,” “r/moreplatesmoredates”). The therapeutic efficacy of GLP-1RAs in some of these populations has been suggested; for example, a recent study showed GLP-1RAs may be a novel treatment target in alcohol use disorder through stimulation of alcohol-related reward centers in the brain.^15^ GLP-1RAs have also been investigated in small studies for PCOS, which was the 6th most popular subreddit overall and is well known to have comorbidities of metabolic syndrome and insulin resistance.^16^ While this has been evaluated in small studies previously,^17^ our study identified blogs which predate the recent American Heart Association Cardiovascular-Kidney-Metabolic Health Presidential Advisory that recognize PCOS as a risk-enhancing factor for which GLP-1RAs may be considered.^18^ The prevalence of posts in these communities suggests individuals with these conditions are interested in GLP-1RAs to improve their health. AI-based social media analysis may serve as a leading indicator to the utility of larger clinical trials in some of these populations to further investigate online anecdotes of suggested benefits.

In contrast, we also identified communities interested in the cosmetic effects of weight loss attributed to GLP-1RA use, such as the bodybuilding (“r/SemaglutideandGains”), reality television (“r/KUWTKsnark,” “r/realhousewives”), and influencer (“r/NYCinfluencersnark”) subreddits. Notably, there were no cardiovascular disease-related subreddits or AI-generated topics identified in our analysis of Reddit despite much excitement about these effects in the medical community. It could be that the population that manifests cardiovascular disease, mostly older adults, are not in the demographic that use Reddit (mostly 18-29 years) and hence are not well represented.^19^ There was also a dearth of references to medical or societal guidelines, suggesting that the medical community may seek to re-examine how best to engage patients through the avenues they seek education about novel health interventions, such as on social media.

We used a pretrained sentiment model called RoBERTa which has been used previously to study health care topics on social media. Most posts were classified as negative or neutral, as was the case in a prior study that investigated statin therapy on Reddit using the same model;^6^ however, our examination suggests that this model may be missing context. To evaluate this further, we randomly selected 9 positive posts and 9 negative posts by RoBERTa and ran these through a more state-of-the-art sentiment model, OpenAI’s gpt-turbo-3.5. Based on this limited sample, it seems RoBERTa classified posts as negative when classically negative terms were mentioned (such as “gambling,” “addiction,” “side effects,” “diabetes,” and “loss”/”losing”), potentially missing context if these conditions are being treated and improving, or if weight loss is occurring, which is the goal of treatment and should be positive. Likewise, if someone is simply asking a question about a negative term (eg “what side effects should I expect?”), this may be more appropriately classified as neutral rather than negative. The OpenAI model seemed to make fewer such errors on this limited sample. As aforementioned we are not able to analyze the entire data set using OpenAI as this is outside the scope the current study, but it is a future direction for research.

### Study Limitations

Our study was restricted by the implementation of scrapping limits during our analysis which prevented us from analyzing Reddit comments or other social media sites, such as X. Incorporation of upvotes, comments, replies, likes, and reposts, perhaps with a weighting mechanism, may provide interesting insights to assess public preferences in future studies. We are also unable to capture posts with spelling errors. Furthermore, while articles are often shared on Reddit, our method of post scrapping did not return many news articles, most likely because the body of these posts did not contain any text.

Our AI clustering was not intuitive and required human review to interpret features the model used to form hierarchies. A standardized way to select number of topics would be preferred. While we attempted to use visualization methods such as the similarity matrix and spatial representation, further work into model interpretability is required. Likewise, we demonstrated RoBERTa may not appropriate for medical topics without further training. In the future, we would like to explore newer large language models, which may have higher performance and be able to perform a more nuanced sentiment analysis. A limited analysis of ChatGPT did appear to perform better on a small sample of our data set. This suggests that exploring other large language models may be more fruitful than to dedicate manpower and time to annotating Reddit posts as ground truth to augment RoBERTa. Chatbots also provide a way to interpret criteria used for sentiment determinations, using a query such as “explain why you classified this post as x sentiment.” We hope to investigate this and other language models in future work, as they also become more affordable and accessible. Incorporating health belief models into our analysis may also help better elucidate public beliefs and needs.

It is incorrect to assume opinions on social media represent the general population, as users are predominantly young adults.^20^ A recent estimate of Reddit suggested only 7% of users are over the age of 50 years.^19^^,^^21^ Furthermore, we have no way to distinguish “bots,” or robot accounts, from real humans and no way to assess the demographics (age, sex, etc) of our study population. The potential for agent provocateurs, either real or bot accounts, to sow discord among the public with controversial or sensational posts is another important area of future study.

## Conclusions

This study demonstrates the potential for AI to help elucidate patterns and themes among social media posts about medications, in this case, GLP-1RAs. Common themes included success stories of improving diabetes and obesity management, struggles with insurance coverage, and questions regarding diet, side effects, and medication administration. Several potential applications include identification of common barriers to medication use, questions and misconceptions that can be addressed in patient-clinician discussions and public health messaging, and the identification of groups who are interested in using these medications for off-label indications to improve their health that may warrant further study. Social media represents a novel epidemiologic data source that can be tapped into using AI; the implications of this on public health warrant further exploration.

Perspectives **COMPETENCY IN INTERPERSONAL AND COMMUNICATION SKILLS:** This article used an AI method to categorize trends in social media posts about GLP-1RA. Posts fell under 3 main themes: insurance and cost issues, diabetes and diet, and medication-specific blogs. Common topics included medication price and insurance coverage challenges, questions about storing pens in the fridge and needle size, weight loss and A1c success stories, and diets to use with GLP-1RAs. In addition to on-label uses for diabetes and obesity, we identified many communities interested in off-label uses such as for PCOS, addictive disorders such as alcohol use or binge eating, and for cosmetic purposes. In conclusion, AI analysis of social media posts gives an unfiltered look at patient experiences, desires, and questions regarding medications which may be used to better inform clinical practice through patient-clinician discussions and early identification of barriers to medication use.

**TRANSLATIONAL OUTLOOK IMPLICATIONS:** Further work is needed to determine how best to apply insights from social media trends about medications, such as GLP-1RAs, to improve patient care.

## Funding support and author disclosures

Under a license agreement between Corrie Health and Johns Hopkins University, the university owns equity in Corrie Health. The university, Drs Marvel and Martin are entitled to royalty distributions related to Corrie Health. In addition, Drs Marvel and Martin are cofounders of and hold equity in Corrie Health. This arrangement has been reviewed and approved by Johns Hopkins University in accordance with its conflict of interest policies. Drs Marvel and Martin have also received research and material support from 10.13039/100017567Apple. Furthermore, Dr Martin is on the Advisory Board for Care Access and reports personal consulting fees from Amgen, BMS, AstraZeneca, Chroma, Kaneka, NewAmsterdam, Novartis, Novo Nordisk, Premier, Sanofi, and 89bio; and also reports research support from the 10.13039/100000968American Heart Association (20SFRN35380046, 20SFRN35490003, #878924, #882415, #946222), the 10.13039/100006093Patient-Centered Outcomes Research Institute (ME-2019C1-15 328, IHS-2021C3-24147), the 10.13039/100000002National Institutes of Health (NIH) (P01 HL108800 and R01AG071032), the 10.13039/100015926David and June Trone Family Foundation, the Pollin Digital Innovation Fund, Sandra and Larry Small, Google, and Merck. All other authors have reported that they have no relationships relevant to the contents of this paper to disclose.
