# Supplementary material for: Trends in Glucagon-Like Peptide-1 Receptor Agonist Social Media Posts Using Artificial Intelligence
Source: JACC Adv. 2024 Aug 28;3(9):101182. doi: 10.1016/j.jacadv.2024.101182 (PMC11450939; doi:10.1016/j.jacadv.2024.101182)
Supplement: Supplemental Data [file mmc1.pdf]

Supplement

All social media posts and the code for all models can be found at: https://github.com/aamirjavaidmd .

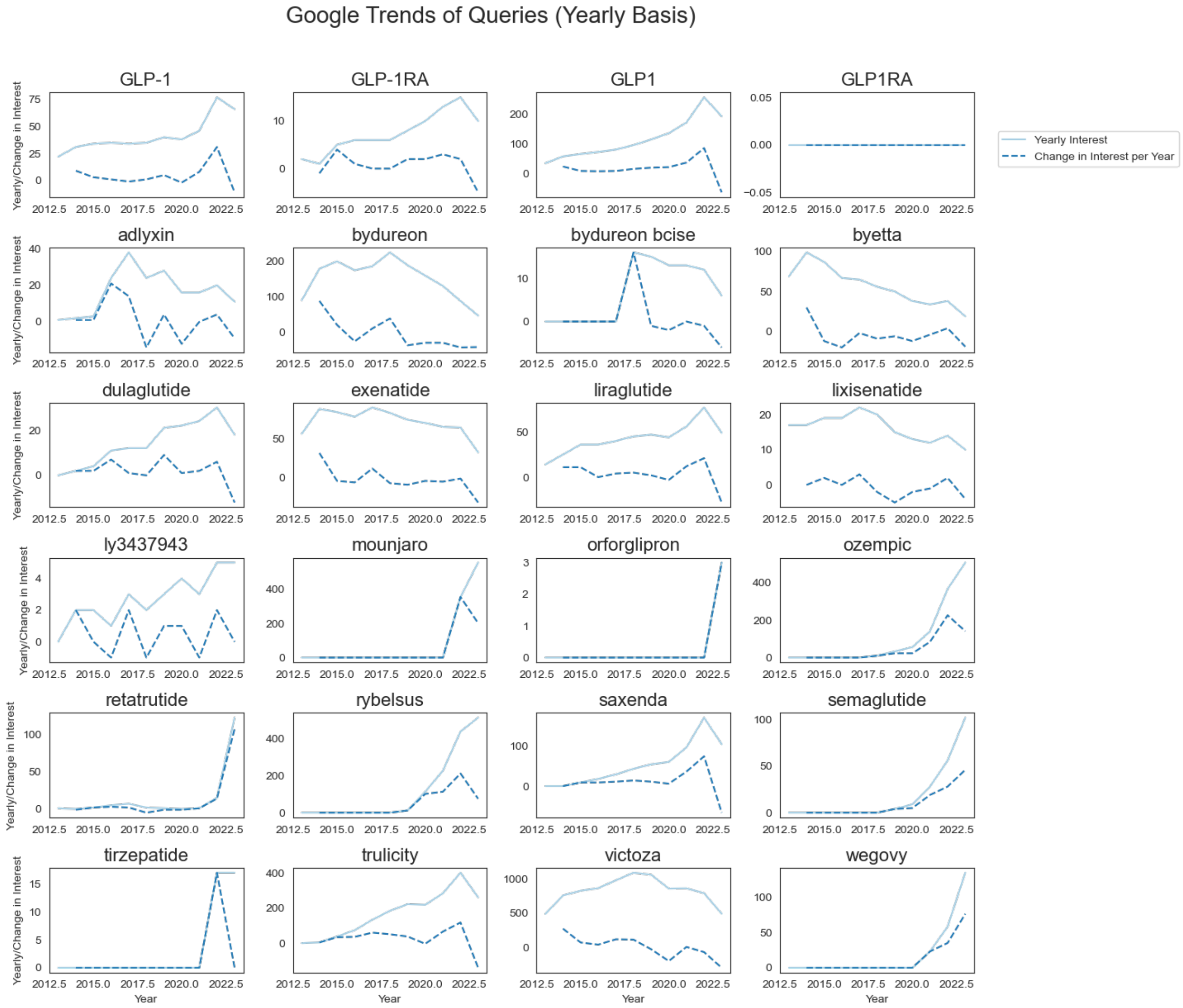

Supplemental Figure 1: Search interest and change in interest per year for each search query on Google Trends.

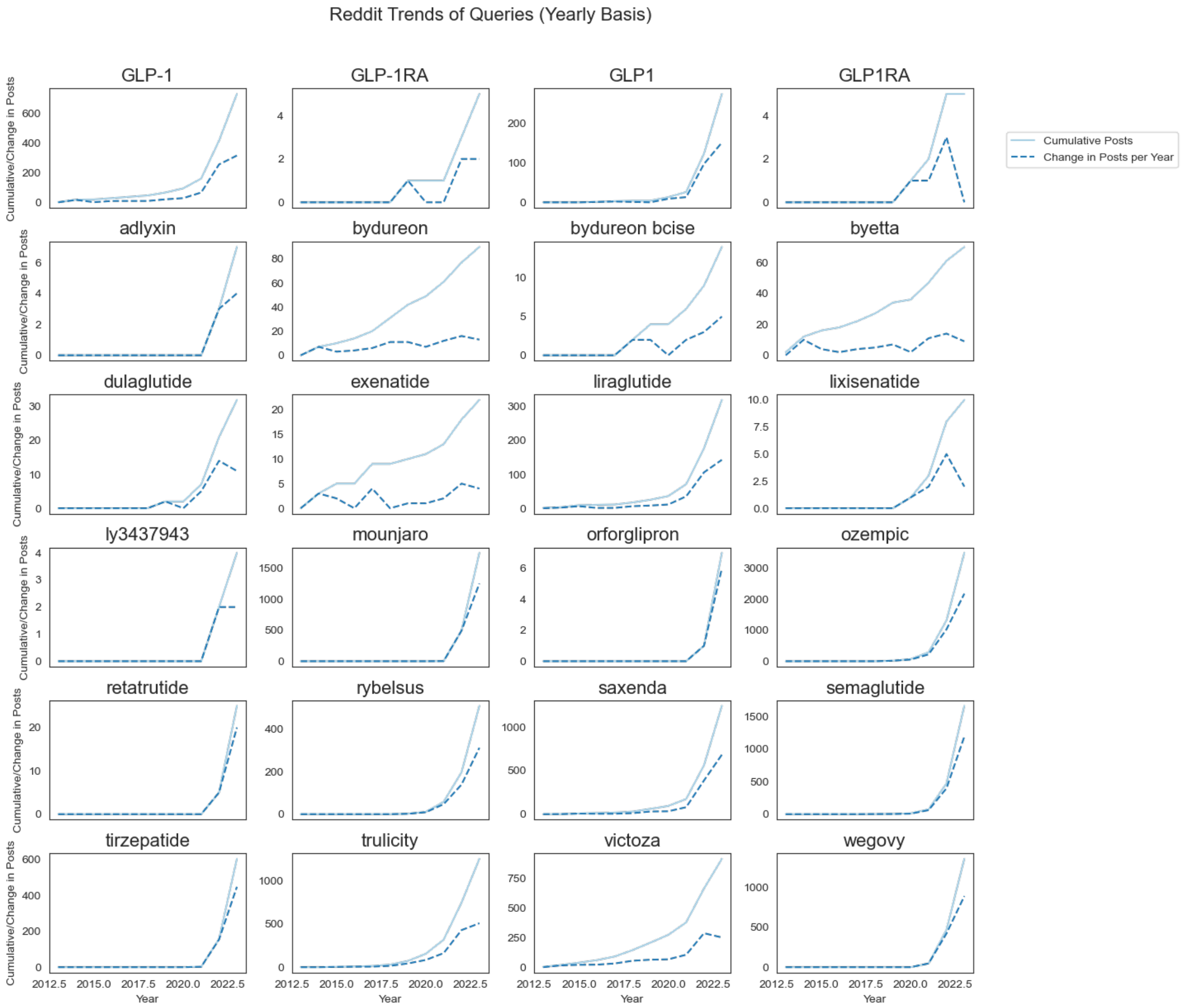

Supplemental Figure 2: Cumulative posts and change in posts per year for each search query group on Reddit.

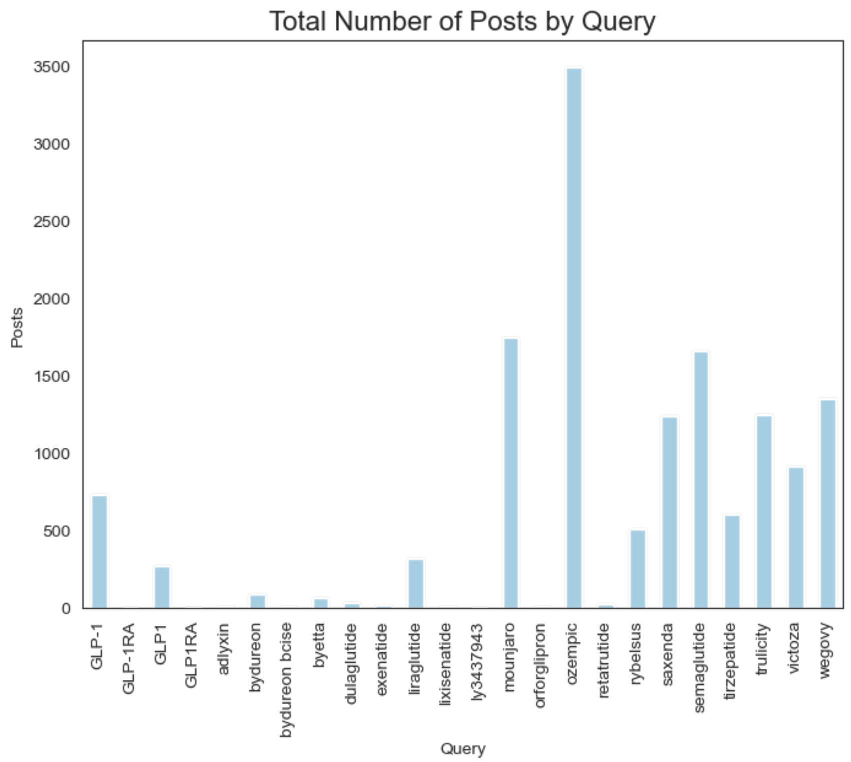
Total Number of Posts by Query
Posts
3500
3000
2500
2000
1500
1000
500
0
GLP-1
GLP-1RA
GLP1
GLP1RA
adlyxin
bydureon
bydureon bcise
byetta
dulaglutide
exenatide
liraglutide
lixisenatide
ly3437943
mounjaro
orforglipron
ozempic
retatrutide
rybelsus
saxenda
semaglutide
tirzepatide
trulicity
victoza
wegovy
Query

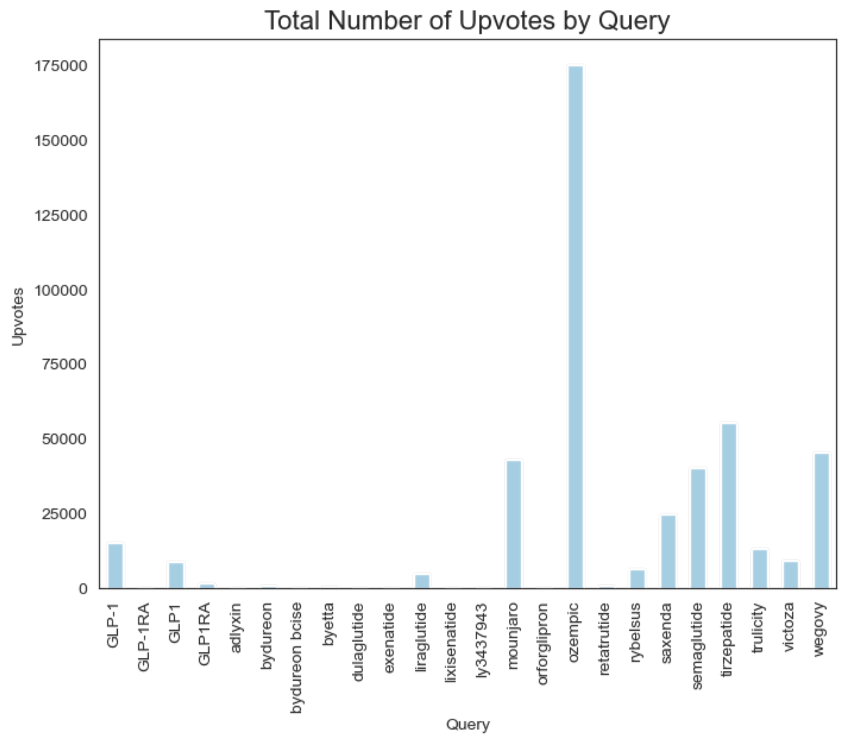
Total Number of Upvotes by Query
Upvotes
175000
150000
125000
100000
75000
50000
25000
0
GLP-1
GLP-1RA
GLP1
GLP1RA
adlyxin
bydureon
bydureon bcise
byetta
dulaglutide
exenatide
liraglutide
lixisenatide
ly3437943
mounjaro
orforglipron
ozempic
retatrutide
rybelsus
saxenda
semaglutide
tirzepatide
trulicity
victoza
wegovy
Query

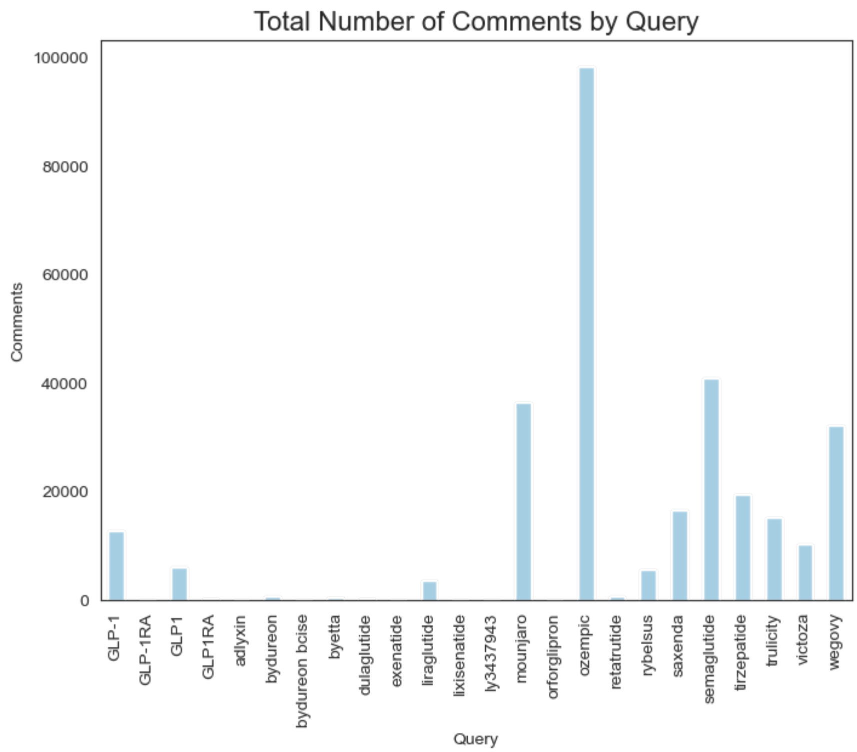

Supplemental Figure 3: Total number of posts, comments, and upvotes by search query.

Supplemental Table 1: number of posts and mean sentiment within each subreddit by RoBERTa.

subreddit	count	mean	std
**Mounjaro**	1445	-0.50796	0.54389
**Semaglutide**	1177	-0.41037	0.527149
**Ozempic**	1090	-0.42569	0.533953
**diabetes**	933	-0.45338	0.521246
**liraglutide**	862	-0.37355	0.534215
**PCOS**	814	-0.46806	0.534964
**diabetes_t2**	693	-0.35931	0.558089
**WegovyWeightLoss**	689	-0.46009	0.53802
**calibrateweightloss**	661	-0.58699	0.548066
**loseit**	514	-0.52918	0.537234
**Tirzepatide**	484	-0.29545	0.512272
**OzempicForWeightLoss**	423	-0.41844	0.521882
**Peptides**	324	-0.30556	0.5
**trulicity**	253	-0.50988	0.531639
**type2diabetes**	215	-0.46977	0.570102
**Mounjaro_ForType2**	209	-0.44976	0.641971
**BingeEatingDisorder**	192	-0.60938	0.510123
**pharmacy**	183	-0.51366	0.51203
**PCOSloseit**	180	-0.49444	0.564274
**moreplatesmoredates**	159	-0.55346	0.523472

**semaglutidecompounds**	152	-0.27632	0.477263
**diabetes_t1**	132	-0.54545	0.529491
**KUWTKsnark**	130	-0.74615	0.436894
**gastricsleeve**	127	-0.55118	0.559317
**SuperMorbidlyObese**	121	-0.47107	0.592655
**NYCinfluencersnark**	118	-0.73729	0.49662
**TheMorningToastSnark**	97	-0.73196	0.549914
**saxendawegovymounjaro**	97	-0.29897	0.482289
**redscarepod**	95	-0.62105	0.549254
**medicine**	92	-0.6413	0.482246
**CommercialsIHate**	89	-0.76404	0.50051
**Testosterone**	89	-0.62921	0.485752
**CourtneyShieldsSnarks**	79	-0.89873	0.343248
**ketoscience**	72	-0.83333	0.375293
**InsulinResistance**	66	-0.45455	0.55972
**Type1Diabetes**	59	-0.35593	0.517378
**RoBody**	58	-0.2931	0.592604
**tinxsnark**	57	-0.54386	0.536862
**GLP1_Ozempic_Wegovy**	49	-0.42857	0.540062
**SemaglutideTalk**	47	-0.17021	0.480903
**CVS**	46	-0.52174	0.505047
**trt**	46	-0.63043	0.488021
**MaintenancePhase**	42	-0.52381	0.551632
**realhousewives**	42	-0.85714	0.354169
**LAinfluencersnark**	38	-0.81579	0.392859
**Supplements**	37	-0.72973	0.450225
**PharmacyTechnician**	36	-0.63889	0.542627
**BravoRealHousewives**	35	-0.94286	0.235504
**RHOBH**	34	-0.76471	0.430562
**WalgreensRx**	32	-0.65625	0.482559
**glp1**	31	-0.77419	0.425024
**science**	31	-0.45161	0.505879
**vegastrees**	31	-1	0
**AarynWilliams**	28	-0.96429	0.188982
**FoodieBeauty**	28	-0.78571	0.417855
**CICO**	27	-0.2963	0.608581
**Menopause**	27	-0.55556	0.50637
**PiaBaronciniSnark**	27	-0.7037	0.465322
**fatlogic**	26	-0.88462	0.325813
**EDAnonymous**	24	-0.70833	0.464306
**notskinnybutnotfat**	24	-0.95833	0.204124

**walmart_RX**	24	-0.375	0.575779
**Health**	22	-0.45455	0.509647
**progresspics**	22	-0.18182	0.58849
**biotech**	21	-0.42857	0.507093
**Peptidesource**	20	-0.35	0.587143
**SemaglutideCompound**	19	-0.21053	0.535303
**Retatrutide**	17	-0.47059	0.514496
**stopdrinking**	17	-0.41176	0.5073
**BIOR**	13	-0.61538	0.50637
**Victoza**	13	-0.23077	0.438529
**CompundedSemaglutide**	11	-0.18182	0.40452
**SourcingSemaglutide**	11	-0.27273	0.467099
**slatestarcodex**	11	-0.63636	0.504525
**GLP1RA**	10	-0.2	0.421637
**Mounjaroadvice**	10	-0.5	0.527046
**longevity**	10	-0.5	0.527046
**mounjaro_ozempic_road**	10	0	0
**SemaglutideandGains**	8	-0.625	0.517549
**canada**	8	-0.5	0.534522
**CanadianOzempic**	7	-0.14286	0.377964
**MounjaroMaintenance**	7	-0.57143	0.534522
**OzempicAustralia**	7	-0.14286	0.377964
**mounjaroweightloss**	7	-0.42857	0.534522
**semaglutideweightloss**	7	-0.57143	0.534522
**EverythingScience**	6	-0.16667	0.408248
**Semaglutide_UK**	6	0	0
**glp1peptides**	6	-0.33333	0.516398
**orforglipron**	6	-0.66667	0.516398
**WeightLossSemaglutide**	4	0	0
**mounjaromeals**	4	0.25	0.5
**MindfulMounjaro**	3	0	0
**triggeredbrandpeptide**	3	0	0
**GLP1Revolution**	2	0	0
**OzempicSellers_USA**	2	0	0
**Ozempic_USA_Source**	2	0	0
**Ozempic_suppliers**	2	0	0
**WegovyWeighIn**	2	-0.5	0.707107
**mounjaro_moms**	2	0	0
**rad140**	2	0	0
**1000lbbestfriends**	1	0	
**GLP1Circlejerk**	1	-1	

**LY3437943**	1	0	
**Mazdutide**	1	-1	
**MounjaroSnark**	1	0	
**MyJuniper**	1	0	
**baseballcards**	1	-1	
**bydureon**	1	0	
**dulaglutide**	1	0	

Supplemental Table 2: number of posts and mean sentiment for each search query by RoBERTa.

query	count	mean	std
**GLP-1**	730	-1	0
**GLP-1RA**	5	-1	0
**GLP1**	274	-1	0
**GLP1RA**	5	-1	0
**adlyxin**	7	-0.85714	0.377964
**bydureon**	90	-0.41111	0.517002
**bydureon bcise**	14	-0.5	0.518875
**byetta**	70	-0.41429	0.524552
**dulaglutide**	32	-0.5	0.508001
**exenatide**	22	-0.27273	0.550482
**liraglutide**	320	-0.39688	0.538769
**lixisenatide**	10	-0.5	0.527046
**ly3437943**	4	-0.25	0.5
**mounjaro**	1748	-0.43764	0.561197
**orforglipron**	7	-0.42857	0.534522
**ozempic**	3491	-0.46061	0.549898
**retatrutide**	25	-0.48	0.509902
**rybelsus**	509	-0.33792	0.532189
**saxenda**	1243	-0.44811	0.533426
**semaglutide**	1662	-0.40794	0.519004
**tirzepatide**	603	-0.41294	0.512597
**trulicity**	1252	-0.45607	0.550114
**victoza**	916	-0.44651	0.535493
**wegovy**	1352	-0.39793	0.528894

Supplemental Table 3. Randomly selected posts classified positive sentiment by RoBERTa compared to ChatGPT sentiment analysis.

Reddit Post	RoBERTa	ChatGPT

Saxenda - a positive side effect for me. After 20 years of chronic diarrhea, Saxenda appears to have cured it. You have no idea how life-changing that is. I no longer have to plan my day around my bathroom habits. I hope it lasts! I've only been on it for 10 days at a low dose, so we will see what happens.	positive	positive
My Eating Disorder got so much better,since I'm medicated (ADHD and Insulin Resistance/Diabetes). My BED got so much better now since I've been diagnosed and medicated for my ADHD. My diabetologist also prescribed me a new medication,a weekly injection called Trulicity.(heard ozempic should be even better) I'm on that (+Metformin) and taking Ritalin now and my appetite and sugar cravings are literally gone. It's crazy how physical my BED actually really was and that is not something i can easily control that much,no matter how much i've been in recovery and try to reduce my emotional eating habits. I struggle with rebound yes,so its a bit risky and i have to pay more attention to it now,cause i tend to not eat enough through the day,but I try to eat a little bit,even if I'm that not hungry. Because at night my hunger comes back,but it's mostly the last 3 days before my new injection where it got worse and out of hand and got "binge attacks" again but they aren't as bad as they were. I didn't buy much sugary foods anymore, cause I'm not craving it and want to keep an eye on my diabetes now. I find some sugar free alternatives for me and everything got better for me now,I track my food ,to keep in check and also try to reach my protein intake,my pcos symptoms got better and I'm losing weight now. I wanna share this to spread awareness that your BED isn't only an emotional thing,it can have multiple factors, i wasn't aware of that! See with the right help (medication) things getting easier for you. For Anyone who suspects to have ADHD and/or Diabetes/Insulin Resistance... Seek a proper diagnosis if thats possible and talk with ur Doctor about medication,it helps a lot!	positive	positive

Weight Gain Frustrations.. T1D for 38yrs. Been through the war & back starting in the 80s with T1D when we had no pumps or CGMs. Struggled with weight gain my whole life. Im 39F. Doesnt help I live in a city where everyone is beautiful & the pressure to be thin & beautiful, has always been fierce. That never bothered me. 2yrs ago I set out in a journey to get fit. Push passed limitation from old injuries etc. lost 25lbs. My ENDO put me on Ozempic. Before anyone steps on the soapbox, please understand my ENDO put me on it. It significantly helped reduce my insulin consumption & manage my glucoses. It was heaven sent. I did all the right things. Watched my diet. Worked out like an animal. In the last 2-3months, I cant get on Ozempic because of the craze over it. I,ve gained about 10ths back. It,s been difficult to co tell my glucoses. I feel really defeated. Any advice ? Anyone gone through this ?	positive	negative
I've got Ozempic Face!. Tongue in cheek title. I haven't seen family for while as I moved to a new country last year. I saw them at the weekend for a family wedding and someone said "have you lost weight? Your face is so skinny!" Level Ozempic Face: unlocked	positive	positive
PA APPROVED! UMR/RxBenefits/CVS Caremark. I just got the message from my Doctor that my insurance company FINALLY approved my PA for mounjaro!!! I’ve been in tears I,m so happy. I had previously had a PA denied and had to go through step therapy. I had to take 3 approved medications (ozempic, trulicity and rybelsus) and fail them before I would get approved. Please don’t give up if you’re facing similar obstacles!! For reference, I’m T2D (6.5 A1C), have PCOS and obesity. I’ve been on a sample of mounjaro for three weeks and it’s changed everything for me. I just had to share with people who would understand this feeling!!	positive	positive
67 Pounds and 8 Months Later!. SW:337 CW:270 GW:230 Y’all, I am celebrating this photo comparison so hard right now! I was on Mounjaro from September thru December, and switched to Saxenda in January due to supply issues. Just started back on 5mg of Mounjaro. Gonna work back up to 7.5mg and see how things go. Here’s to 67 pounds gone and only 40 more to go! This medicine has changed my life, seriously! I’ve stopped drinking and smoking and no longer struggle with my eating disorders (BED and ANNA). For the first time in my life I feel normal Saxenda was ok, but pales in comparison to MJ! So ready to restart this thing and tackle the summer and my health head on! Cheers!	positive	positive

Normal A1C after 2 months!. A year ago, my A1C was 11. I got it down to 8. When I went to my doc in January, it was 8.3. I was just happy it wasn't 11 again. She prescribed Mounjaro 10 to start. Pharmacy couldn't get 10. After few weeks, she changed it to 7.5. That wasn't strong enough, so toward the end of February I started on 12.5. I had a followup with my doctor yesterday. My A1C was 6.4!!! This drug, I just can't say enough good things about it. I took Victoza injections daily for years and never got the results I am getting with Mounjaro.	positive	positive
Exciting Follow Up. Ahhhhh!!!! I just got out of my follow up appointment after first being diagnosed on January 26th!!!! And I got the best news EVER! I WAS ABLE TO BRING MY A1C FROM A 10.3 TO A 5.7!!!! I'm so happy! My doctor is taking me off trulicity and now I'll only be take a 500 dose of metformin once a day!	positive	positive
Switching to Ozempic. ****TWO-WEEK UPDATE**** It's been two weeks, and I've lost 7 lbs, which is AWESOME! The side effects have been tolerable so far, with only slight nausea on the first day after taking my dose. Other than that, I feel alright. The appetite suppressant effect seems stronger than what I experienced with Trulicity. Has anyone switched to Ozempic from Trulicity? What's been your experience? I'm nervous. I'm hoping I don't have crazy side effects!	positive	positive

Supplemental Table 4. Randomly selected posts classified negative sentiment by RoBERTa compared to ChatGPT sentiment analysis.

Reddit Post	RoBERTa	ChatGPT
Mounjaro and other addictions.. Today I saw a report of Ozempic having also stopped some addiction in highly addicted people such as smoking, drinking etc. I do not take Ozempic but do take Mounjaro for past 11 weeks which also subconsiously unknowingly for me happens to be how long I stopped gambling, and that was an unstoppable urge for past 20 years. I thought the 18lb loss was a miracle but the poker gambling is such a bonus.	negative	positive

Already Giving Up. hi, i just got prescribed Mounjaro a day ago by my primary care doctor. I am pre-diabetic and insulin resistant with A1C of 6.1. The pharmacy said my insurance would not cover it and they didn't not explain why. I reached out to my insurance and she said that the reason why it was not approved because I don't have a T2D diagnosis. She made it seem like I either need a prior authorization, step therapy, AND a T2D diagnosis to get this medication. I checked the updated formulary and I only seen Ozempic and Mounjaro covered. I've seen people on this sub get it covered without a T2D diagnosis but it seems unlikely. I have BCBS select silver plan and I asked my doctors office to send in a prior authorization and step therapy (I've taken metformin but the side effects were terrible) I know it takes time for a PA and such but I'm just scared what the next steps would be if I can't get it covered. On another part of my insurance under a form labeled maintenance drugs with insulin said other drugs like Saxenda and Wegovy were covered, but I believe this only means if you are taking insulin.	negative	negative
Looks like its over for me?!. Well, I like everyone else it seems right now, am having an EXTREMELY hard time getting my mounjaro refill. I am looking at switching to another medication, does anyone know of another weight loss medication that has a coupon? OR does anyone have insurance that covers weight loss meds? I am in the process of switching my insurance and might be able to pick one that covers weight loss meds.... Thanks for any and all help!!	negative	negative
PA denials. My insurance isn't budging on the PA because even though my fasting sugars are outrageous without being on MJ/Tirzepatide my A1C has never been 7+ It was 6.4 in my last work up. Anyone ever get theirs approved and had this issue, if so how? Thanks	negative	neutral
PA has been approved. I just got my PA approved, and my insurance will cover all but $25 for three months supply!! I'm so excited. I am switching from Ozempic to Mounjaro. Is there much difference in the side effects? Of the severity of the side effects? Thanks :)	negative	positive
what's best to take for weight loss. I'm 38f 180 looking to lose weight. What's the best supplement for losing weight.	negative	neutral

Ozempic in pill form?. Our GP has just told my sister that Ozempic now existed in pill form... she can't inject so she's beyond excited. Would anyone know the name of that medication? GP forgot (I rest my case). Thanks.	negative	positive
Sick to stomach. Hi. I took my first shot Wednesday. My first side effect was a headache. Then started light heartburn & feeling sick to my stomach. The sick to my stomach is hanging around. Any suggestions??	negative	negative
Obgyn Appointment. Hey everyone! I am going to my annual Obgyn appointment today. I was diagnosed with pcos two years ago and since then haven't had a period, was put on birth control, and am now showing signs of pre diabetes. I am trying to get on ozempic and get a continuous glucose monitor. What are the questions and things I need to tell my Obgyn?	negative	neutral

Supplemental Table 5. Randomly selected posts with discordant sentiment by RoBERTa compared to ChatGPT.

Reddit Post	RoBERTa	ChatGPT
Weight Gain Frustrations.. T1D for 38yrs. Been through the war & back starting in the 80s with T1D when we had no pumps or CGMs. Struggled with weight gain my whole life. Im 39F. Doesnt help I live in a city where everyone is beautiful & the pressure to be thin & beautiful, has always been fierce. That never bothered me. 2yrs ago I set out in a journey to get fit. Push passed limitation from old injuries etc. lost 25lbs. My ENDO put me on Ozempic. Before anyone steps on the soapbox, please understand my ENDO put me on it. It significantly helped reduce my insulin consumption & manage my glucoses. It was heaven sent. I did all the right things. Watched my diet. Worked out like an animal. In the last 2-3months, I cant get on Ozempic because of the craze over it. I,ve gained about 10ths back. It,s been difficult to co tell my glucoses. I feel really defeated. Any advice ? Anyone gone through this ?	positive	negative

Mounjaro and other addictions.. Today I saw a report of Ozempic having also stopped some addiction in highly addicted people such as smoking, drinking etc. I do not take Ozempic but do take Mounjaro for past 11 weeks which also subconsiously unknowingly for me happens to be how long I stopped gambling, and that was an unstoppable urge for past 20 years. I thought the 18lb loss was a miracle but the poker gambling is such a bonus.	negative	positive
PA denials. My insurance isn't budging on the PA because even though my fasting sugars are outrageous without being on MJ/Tirzepatide my A1C has never been 7+ It was 6.4 in my last work up. Anyone ever get theirs approved and had this issue, if so how? Thanks	negative	neutral
PA has been approved. I just got my PA approved, and my insurance will cover all but $25 for three months supply!! I'm so excited. I am switching from Ozempic to Mounjaro. Is there much difference in the side effects? Of the severity of the side effects? Thanks :)	negative	positive
what's best to take for weight loss. I'm 38f 180 looking to lose weight. What's the best supplement for losing weight.	negative	neutral
Ozempic in pill form?. Our GP has just told my sister that Ozempic now existed in pill form... she can't inject so she's beyond excited. Would anyone know the name of that medication? GP forgot (I rest my case). Thanks.	negative	positive
Obgyn Appointment. Hey everyone! I am going to my annual Obgyn appointment today. I was diagnosed with pcos two years ago and since then haven't had a period, was put on birth control, and am now showing signs of pre diabetes. I am trying to get on ozempic and get a continuous glucose monitor. What are the questions and things I need to tell my Obgyn?	negative	neutral
